# Presence of infection in patients with presumed sepsis at the time of ICU admission

**DOI:** 10.1186/cc14066

**Published:** 2014-12-03

**Authors:** PMC Klein Klouwenberg, OL Cremer, LA van Vught, DSY Ong, JF Frencken, MJ Schultz, MJ Bonten, T van der Poll

**Affiliations:** 1 Department of Intensive Care Medicine, University Medical Center Utrecht, the Netherlands; 2 Department of Medical Microbiology, University Medical Center Utrecht, the Netherlands; 3 Julius Center for Health Sciences and Primary Care, University Medical Center Utrecht, the Netherlands; 4 Center for Experimental and Molecular Medicine and Division of Infectious Diseases, Academic Medical Center, University of Amsterdam, the Netherlands; 5 Department of Intensive Care Medicine, Academic Medical Center, University of Amsterdam, the Netherlands

## Introduction

A clinical suspicion of infection is mandatory for diagnosing sepsis in patients with a systemic inflammatory response syndrome. Yet the accuracy of categorizing critically ill patients presenting to the ICU as being infected or not is unknown. We therefore assessed the likelihood of infection in patients who were treated for sepsis upon admission to the ICU, and quantified the association between plausibility of infection and mortality.

## Methods

We studied a cohort of critically ill patients admitted with clinically suspected sepsis to two tertiary ICUs in the Netherlands between January 2011 and December 2013. The likelihood of infection was categorized as none, possible, probable or definite by *post hoc *assessment. We used multivariable competing risks survival analyses to determine the association of the plausibility of infection with mortality.

## Results

Among 2,579 patients treated for sepsis, 13% had a *post hoc *infection likelihood of 'none', and an additional 30% of only 'possible'. These percentages were largely similar for different primary suspected sites of infection. In crude analyses, the likelihood of infection had no impact on ICU mortality, but was associated with increased length of stay and complications. In multivariable analysis, however, patients with an unlikely infection had a higher mortality rate compared to patients with a definite infection (subdistribution hazard ratio 1.23; 95% confidence interval 1.03 to 1.49).

## Conclusion

This study is the first prospective analysis to show that the clinical diagnosis of sepsis upon ICU admission corresponds poorly with the presence of infection on *post hoc *assessment. A higher likelihood of infection does not adversely influence outcome in this population.

